# Role of New Biomarkers: Functional and Structural Damage

**DOI:** 10.1155/2013/361078

**Published:** 2013-02-05

**Authors:** Evdoxia Tsigou, Vasiliki Psallida, Christos Demponeras, Eleni Boutzouka, George Baltopoulos

**Affiliations:** Athens University School of Nursing ICU, “Agioi Anargyroi” General Hospital, Kifisia, 14564 Athens, Greece

## Abstract

Traditional diagnosis of acute kidney injury (AKI) depends on detection of oliguria and rise of serum creatinine level, which is an unreliable and delayed marker of kidney damage. Delayed diagnosis of AKI in the critically ill patient is related to increased morbidity and mortality, prolonged length of stay, and cost escalation. The discovery of a reliable biomarker for early diagnosis of AKI would be very helpful in facilitating early intervention, evaluating the effectiveness of therapy, and eventually reducing cost and improving outcome. Innovative technologies such as genomics and proteomics have contributed to the discovery of new biomarkers, such as neutrophil gelatinase-associated lipocalin (NGAL), cystatin C (Cys C), kidney injury molecule-1 (KIM-1), interleukin-18 (IL-18), and liver-type fatty acid binding protein (L-FABP). The current status of the most promising of these novel AKI biomarkers, including NGAL, Cys C, KIM-1, L-FABP, and IL-18, is reviewed.

## 1. Introduction

Acute kidney injury is a heterogeneous process, defined by the Acute Kidney Injury Network (AKIN) as “functional and structural disorder or signs of renal damage including any defect from blood and urine test, or tissue imaging that is less than 3 months”. AKI is usually classified into three broad categories as follows: (a) prerenal—an adaptive response to severe volume depletion and/or hypotension, with structurally intact nephrons—it is a functional damage that represents the most common form of kidney injury but can lead to intrinsic AKI if it is not promptly corrected; (b) intrinsic—structural injury in the kidney, which is the hallmark of intrinsic AKI—the most common form of intrinsic injury is acute tubular necrosis (ATN), either due to ischemic, inflammatory, or cytotoxic insults; and (c) postrenal—from mechanical obstruction of the urinary collecting system, including the renal pelvis, ureters, bladder, or urethra [1].

“RIFLE” (Risk, Injury, Failure, Loss, and End-Stage Kidney Disease) and AKIN criteria are the most frequently used scoring systems [1, 2]. Therapeutic interventions have failed to reduce morbidity and mortality, largely due to the heterogeneity of the process, the incomplete understanding of AKI pathogenesis, and therefore the delayed diagnosis and implementation of the various procedures. The reliance on conventional biomarkers such as urea and creatinine explains the time frame between the occurrence and detection of the disease process [3].

AKI biomarkers can be components of serum or urine. The term biomarker (acronym for biological marker) was first described in 1989, which means measurable indicator for a specific biologic condition and for specific disease process. In 2001, biomarker definition was standardized to be a characteristic that can be measured and evaluated as a normal biological process, pathological process, or pharmacological response to therapeutic intervention. Moreover, the Food Drug and Administration (FDA) uses the biomarker term to describe any diagnostic indicator that can be measured and used to assess any risk or disease [4].

The ideal renal biomarker should have the following characteristics:

- diagnose renal dysfunction promptly,
- distinguish prerenal AKI from apoptotic and necrotic injury,
- be able to localize the damage (e.g., tubular versus other primary locations),
- be specific for renal injury in the presence of concomitant injury involving other organs,
- be able to discern AKI from chronic kidney disease,
- classify according to severity,
- predict the outcome,
- permit disease modification,
- be inexpensive and easy to perform,
- be able to act as an endpoint, useful for interventional studies [5].

Traditional biomarkers are far away from satisfying the above requirements. Urea production is inconstant, and values can be altered by nonrenal factors such as changes in endovascular volume, protein intake, and presence of gastrointestinal bleeding. Furthermore, 40–50% of filtered urea can be reabsorbed in the tubule [6]. Serum creatinine (SCr) remains the cornerstone of AKI diagnosis. Nevertheless, it has several serious limitations. Its value varies with age, gender, diet, muscle mass, drugs, and vigorous exercise. Creatinine is also secreted by the urinary tubules and this stands for 10–40% of its clearance. So, in cases of decreased glomelural filtration rate (GFR) serum creatinine levels may remain within normal values. Additionally, creatinine becomes abnormal when more than 50% of GFR is lost, and it takes up to 24 hours before increases in blood concentration are detectable [7]. Estimation of GFR is most commonly based upon the serum creatinine concentration: the Cockcroft-Gault equation or the Modification of Diet in Renal Disease (MDRD) equation [8]. 

Innovative technologies such as genomics and proteomics made it possible to discover earlier biomarkers of AKI, including neutrophil gelatinase-associated lipocalin (NGAL), cystatin C (Cys C), kidney injury molecule-1 (KIM-1), interleukin-18 (IL-18), and liver-type fatty acids binding protein (L-FABP). The current status of these novel AKI biomarkers is reviewed.

## 2. NGAL

### 2.1. Biochemical Characteristics and Biologic Properties

NGAL (syn.lipocalin 2, siderocalin) is a small molecule of 178 amino acids that belongs to the lipocalin superfamily of 20 structurally related secreted proteins. It occurs predominantly in a monomeric form, with a small percentage occurring as a dimer or trimer. Lipocalins are characterized by eight *β*-strands that form a *β*-barrel defining a calyx. The calyx binds and transports a wide variety of low molecular weight molecules, which are thought to define the biologic activity of each lipocalin. For example, retinol-binding protein binds and transports vitamin A, the lipocalin a1-microglobulin scavenges heme [36]. 

NGAL, like the other lipocalins, is able to bind some ligands, including the siderophores. Interactions with iron-binding siderophores are responsible for NGAL's characteristic bright red colour and determine its bacteriostatic effects. Bacteria produce siderophores to scavenge iron from the extracellular space and use specific transporters to recover the siderophore-iron complex, ensuring their iron supply. NGAL binds with these siderophores and transports them within cells, after interacting with specific membrane receptors. Among the several membrane proteins, the 24p3 cell-surface receptor (24p3R) and the megalin multiscavenger complex represent the most important molecules. The interaction of NGAL with the receptor leads to the internalization of the complex NGAL-siderophore, producing a significant increase in cytoplasmic iron levels. NGAL can prevent growth of the bacterial strains that rely on the production of siderophores to satisfy their iron demands. The biologic significance of this finding was demonstrated in genetically modified mice, which were deficient for both copies of the NGAL gene. These animals were more sensitive to certain Gram-negative bacteria and more readily died of sepsis than did wild-type mice [37].

Therefore, NGAL represents a critical component of innate immunity to bacterial infection. Actually human NGAL was originally identified as a 25-kD protein covalently bound to gelatinase from human neutrophils, where it represents one of the neutrophil secondary granule proteins. NGAL is expressed at very low levels in human tissues, including kidney, trachea, lungs, stomach, and colon, and its expression increases greatly in the presence of inflammation and injured epithelia [38]. 

NGAL as a renal biomarker was discovered in 2003, following experimental renal ischemia in a mouse model. With the use of transcriptomic approaches seven genes were identified, whose expression was upregulated 10-fold within the first few hours. Among them, lipocalin was the gene with the earliest and highest rise of mRNA and protein concentration in renal tissue, urine, and plasma. NGAL protein expression was detected predominantly in proliferating proximal tubule cells. The rapidity of the process was against the polymorphonuclear origin of NGAL [39].

Both plasma and urine NGALs are increased after a renal insult, and even though the kidney seems to be the major source of elevated plasma lipocalin, there are other plausible explanations. Several studies in human and animal models have demonstrated that AKI results in an increased NGAL mRNA expression in distant organs, especially the liver and lungs, contributing to the increased levels. This effect may further increase urine levels as a result of insufficient reabsorption of the filtered load. Also, NGAL is an acute-phase reactant and may be released from neutrophils, macrophages, and other immune cells. Any decrease in GFR resulting from AKI would be expected to decrease the renal clearance of NGAL, with subsequent accumulation in the systemic circulation [40]. Plasma NGAL is freely filtered by the glomerulus, and it is largely reabsorbed in the proximal tubules by efficient megalin-dependent endocytosis. Thus, any urinary excretion of NGAL is likely only when there is a concomitant proximal renal tubular injury that precludes NGAL reabsorption and/or increase de novo NGAL synthesis. However, gene expression studies in AKI have demonstrated a rapid and massive upregulation of NGAL mRNA in the distal nephron segments—specifically in the thick ascending limb of Henle's loop and the collecting ducts. The resultant synthesis of NGAL protein in the distal nephron and secretion into the urine appears to comprise the major fraction of urinary NGAL [41]. So, elevated urine NGAL originates from both proximal and distal nephron after a nephrotoxic insult. Radiocontrast nephropathy, a prototype of hypoxia-mediated nephrotoxicity with distal tubular injury, leads to overexpression of NGAL [42]. NGAL is also increased in experimental cisplatin toxicity, characterized by injury to the S3 segment of the proximal tubule [43]. It seems to be that NGAL is a common and sensitive response to tubular injury, without differentiating the distribution pattern of tubular injury. 

NGAL, apart from its antibacterial effect via iron sequestration, reduces proapoptotic processes and in this way appears to limit damage to the proximal tubule. Of importance, NGAL regulates intrarenal iron metabolism and acts to stimulate proliferation and epithelialization [44]. It has been speculated that the increase in the NGAL level after renal tubular injury may serve to limit injury in recurrent insults or even ameliorate the degree of damage in an ongoing insult. Many functional roles of NGAL can be explained by complex interactions with iron transporters, including anti-inflammatory actions, embryogenesis, and neoplastic growth [45].

### 2.2. Assay Methods for NGAL Measurement and Reference Values

NGAL was initially measured by manual and not standardized ELISA or immunoblotting systems, methods that are only recommended for research studies [9]. There are commercially available ELISA kits (NGAL Rapid ELISA KIT 037, Antibodyshop ELISA kit, BioPorto Diagnostics) that can be used manually or by chemistry analyzers [46]. There is also available a point-of-care test (POCT) method (Triage Bioste, Alere Health), which is a rapid (30 minutes) fluorescence-based immunoassay [47].

More recently, a CMIA (chemiluminescent microparticle immunoassay) method became commercially available, using the automated platform ARCHITECT (Abbott Diagnostics) for the measurement of NGAL in urine samples [48]. 

Reference values of NGAL are rather arbitrary, since they have emerged from groups of healthy people that participated in a clinical study as a control group. As an example, Stejskal et al. observed similar values of serum NGAL in healthy men and women, that is to say, 86.3 ± 43.0 ng/mL and 88.9 ± 38.2 ng/mL, respectively [49]. The expected range of NGAL normal values of Triage NGAL Test is 149 ng/mL, with a 90% confidence interval ranging from 100 to 194 ng/mL [50]. The kit insert of the CMIA assay reports a value of 132 ng/mL as the 95th percentile of NGAL values, measured in 196 blood donors [51]. 

Limitations regarding NGAL measurement include storage conditions (NGAL is stable in urine if stored at 4°C for up to 7 days and plasma or urine samples are stable if stored for a long time at −80°C), presence of haemolysis, and production of NGAL by neutrophils in urinary tract infections. In order to limit this phenomenon, urine should be centrifugated to remove neutrophils in cases of urinary tract infection. Also, plasma measurements are preferable to serum, since some NGALs may be released from neutrophils during the preparation of serum [52].

### 2.3. Performance of NGAL in Various Clinical Settings

Several studies have evaluated serum NGAL (sNGAL) and urine NGAL (uNGAL) as biomarkers of AKI in different patients' populations. AKI is a highly heterogeneous syndrome that involves the complex interaction between vascular, tubular, and inflammatory factors. The susceptibility of kidney to ischemia and toxins can be explained by the vulnerability of tubular cells of the outer medulla to ischemia/hypoxia and the exposure of local epithelial cells to substances that are filtered and reabsorbed by the nephron [53].

### 2.4. NGAL in Cardiac Surgery

Acute renal dysfunction is very common in adult cardiac surgery patients, and it is associated with a mortality that approximates to 80%. The diminished renal blood flow during the procedure, the loss of pulsatile flow, the hypothermia, and the intraoperative inflammatory response have all been implicated in the pathogenesis of the syndrome. AKI is less common in infants and children with congenital heart disease submitted to surgical repair, due to the absence of comorbidity in the pediatric population. In general, AKI complicates almost 40% of adult and 10% of pediatric cardiac operations, respectively [54]. 

Performance of sNGAL and uNGAL in the setting of AKI that follows cardiac surgery has been investigated in well designed studies in both critically ill children and adults. These studies are summarized in Table 1. 

The study of Misra et al. was one of the first to examine the reliability of NGAL as a prognostic marker of AKI after cardiac surgery for congenital heart defects in children. Twenty of 71 children developed AKI, which was defined as a 50% increase in serum creatinine from baseline. The level of uNGAL at 2 h after cardiopulmonary bypass was the most powerful independent predictor of acute renal injury. For uNGAL at 2 h, the area under the receiver-operating characteristic curve (AUC-ROC) was 0.99, sensitivity was 100%, and specificity was 98% for a cutoff value of 50 ng/mL. pNGAL had a good, though having lower performance than uNGAL, for the diagnosis of AKI. NGAL-based diagnosis of AKI preceded routine diagnosis by serum creatinine by 1–3 days after cardiopulmonary bypass (CPB) [10]. Dent et al. also found an excellent performance of plasma NGAL, at a cutoff value of 150 ng/mL in pediatric cardiac surgery population [11]. Bennett et al., again in pediatric population, showed that uNGAL at a cutoff point of 100 ng/mL had an AUC-ROC of 0.95 [13]. Krawczeski et al. investigated four possible biomarkers in the urine, NGAL, IL-18, L-FABP, and KIM-1 at various time points after CPB in children. Urine NGAL was the first to rise in AKI patients, even at the first 2 hours after CPB initiation. At all time points NGAL maintained the best predictive performance (0.90, 0.91, 0.90, and 0.87 at 2 hours, 6 hours, 12 hours, and 24 hours, resp.) [21]. 

Studies focusing on adult cardiac patients concluded to controversial remarks, with AUC varying from 0.56 to 0.98. Perry et al. in their retrospective study on postoperative coronary artery bypass graft patients found that 8.6% of patients developed AKI and that pNGAL measured immediately after separating from CPB had a sensitivity of only 38.7% (cutoff point of 353.5 ng/mL) [20]. Parikh et al. recently completed a prospective, multicenter study involving 1219 adults undergoing cardiac surgery and evaluated urine IL-18, uNGAL, and pNGAL as possible predictive markers of AKI. All three markers peaked at 6 hours after surgery but had a poor discriminative ability for AKI. Urine NGAL at a cutoff point of 102 ng/mL had a sensitivity of 46% and a specificity of 81%, and plasma NGAL at a cutoff point of 293 ng/mL had a sensitivity of 50% and a specificity of 82%. The AUC-ROC was 0.67 and 0.70, respectively. On the other hand, the clinical model for AKI had an AUC of 0.69, and pNGAL significantly improved the AUC to 0.75, whereas uNGAL did not improve the AUC above that of the clinical model [22].

Possible explanations for the inferior performance of NGAL in adults comparatively to children may be the older age and the presence of comorbidities, especially preexisting kidney disease. Different timing of NGAL measurement in relation to the renal insult and different specimen preparation or measuring techniques or storage may in part explain the variability of the results in adults. Nevertheless, in most cardiac surgery studies, NGAL concentration was proportional to the degree of severity and duration of AKI, and in multivariate regression analyses it was the strongest independent risk factor for AKI [55].

In the typical ICU patients suffering from AKI, the timing of the renal insult is largery unknown. This fact poses obstacles to the interpretation of an elevated NGAL concentration, especially in the presence of sepsis. Studies concerning mixed ICU patients, both children and adults, are presented in Table 2.

Zappitelli et al. in a group of 140 critically ill kids found that uNGAL rose 2 days before serum creatinine, in cases of AKI. Urine NGAL had acceptable characteristics as a diagnostic marker of AKI, with an AUC of 0.79, but was not associated with the severity of renal injury [25]. Wheeler et al. focused on children with systemic inflammatory response syndrome (SIRS) or septic shock. They measured serum NGAL during the first 24 hours of admission to the ICU. They concluded that sNGAL could discriminate between healthy children, critically ill children with SIRS, and critically ill children with septic shock. Serum NGAL was significantly increased in critically ill children with AKI compared with those without AKI [26]. 

In a heterogeneous population of 451 critically ill adults, Siew et al. found urine NGAL to have a moderate ability for the diagnosis of AKI at 24 and 48 h from enrolment. Median uNGAL levels were significantly higher in the subjects who died, as well as in the subjects receiving acute dialysis [28]. Cruz et al. enrolled 307 consecutive adult patients admitted to a general medical surgical ICU and found that plasma NGAL measured at ICU admission was a good diagnostic marker for AKI development within the next 48 h with an AUC of 0.78. Plasma NGAL was also a reliable parameter for renal replacement therapy (RRT) use, with an AUC of 0.82 [30]. De Geus et al. showed that both pNGAL and uNGAL measured at ICU admission do not outstrip serum creatinine-derived GFR for the prediction of severe AKI. However, NGAL added significant accuracy to this prediction in combination with GFR alone or with other clinical parameters [34]. 

The role of NGAL as an inflammatory biomarker might hamper this discriminative ability in cases of septic AKI, which is actually the most common in critically ill patients. Bagshaw et al. investigated if pNGAL and uNGAL could differentiate between septic and nonseptic AKI, and they found that septic AKI was associated with significantly higher plasma and urine NGAL at enrolment, compared to non-septic AKI [56]. On the other hand, Mårtensson et al. showed that pNGAL could not discriminate between septic shock and AKI, whereas uNGAL was a good predictor for AKI within the next 12 hours in patients with septic shock [32].

The correlation of NGAL with the severity of AKI was examined by Haase-Fielitz et al. who showed in 100 cardiac ICU patients that the discriminatory ability of NGAL for AKI increased with increasing RIFLE classes or AKIN stages. It was even highest for the prediction of renal replacement therapy (AUC-ROC: 0.83) [57].

### 2.5. Other Roles of NGAL in ICU Patients

Performance of NGAL as a predictor of AKI in the emergency department (ED) has been examined by Nickolas et al. who measured uNGAL on 635 consecutive ED patients and found that it had excellent sensitivity and specificity (90 and 99% at a 130 mg/g creatinine cutoff), as well as the ability to differentiate between AKI and other causes of elevated creatinine, such as chronic kidney disease and prerenal azotemia [58].

NGAL has been evaluated as a biomarker of delayed graft function (DGF, defined as dialysis requirement within the first postoperative week) in patients undergoing kidney transplantation. In prospective multicenter studies, urine NGAL levels in samples collected on the day of transplant identified those who subsequently developed DGF with an AUC of 0.8–0.9 [59, 60]. 

NGAL is also emerging as an early biomarker in interventional trials. For example, the response of urine NGAL was attenuated in adult cardiac surgery patients who experienced a lower incidence of AKI after sodium bicarbonate therapy when compared to that after sodium chloride [61]. 

NGAL proved to be of a good prognostic value in the prediction of the need for RRT initiation or mortality. Dent et al. found pNGAL to be a reliable predictor of duration of AKI and length of hospital stay, while the 12-hour pNGAL level was a predictor of mortality [11]. Similarly, Bennett et al. found the 2-hour uNGAL to be a reliable predictor of severity and duration of AKI, length of hospital stay, RRT requirement, and mortality in 196 children undergoing CPB [13]. Kümpers et al. evaluated the predictive value of serum NGAL in 109 patients with established AKI at inception of RRT in the ICU. They found a significant difference in serum NGAL between healthy subjects, critically ill patients with SIRS, and critically ill patients with sepsis. NGAL was an independent predictor of 28-day mortality, with an AUC of 0.74. NGAL levels were independently related to the severity of AKI and the extent of systemic inflammation [62]. The finding that blood NGAL is not substantially cleared by continuous veno-venous hemofiltration support the use of NGAL as an early indicator of renal recovery in critically ill patients supported by renal replacement therapy. 

Haase et al. published a meta-analysis for NGAL, including 24 studies and 2538 patients. They analyzed both urine and plasma/serum NGAL studies with measurements within 6 h of renal insult or from 24 to 48 h before the diagnosis of AKI by conventional means. They demonstrated that the overall diagnostic odds ratio for NGAL to predict AKI was 18.6 with an AUC of 0.815, sensitivity of 76.4%, and specificity of 85.1%. The results were slightly better for children than adults and improved when studies used standard assays for NGAL detection rather than research-based ones. Notably in cardiac surgery cases, NGAL had a diagnostic odds ratio of 13.1 with an AUC of 0.775 and a sensitivity and specificity of 75.5 and 75.1%, respectively. uNGAL was found to be slightly superior to plasma measurements in the meta-analysis with an AUC of 0.837 versus 0.775 for plasma or serum. NGAL also correlated fairly well with predication of renal replacement therapy initiation with an AUC of 0.782, but not with in-hospital mortality [63]. The properties of NGAL as an AKI biomarker, as well as the properties of the other evaluated biomarkers, are briefly presented in Table 3.

## 3. Limitations of NGAL as a Biomarker of AKI

Although NGAL has been highlighted as a reliable biomarker of AKI in critically ill patients, its use is not without limitations. First of all, plasma NGAL measurements may be influenced by a number of coexisting variables, including chronic kidney disease, chronic hypertension, systemic infections, inflammatory conditions, anemia, hypoxia, and malignancies. In cases of anemia there is an increase in the peripheral production of NGAL, in order to counteract the hypoxic stress. On the other hand NGAL can suppress erythropoiesis and worsen anemia [64]. In chronic kidney disease NGAL levels correlate with the severity of renal impairment. However, it should be noted that the increased plasma NGAL in all the above situations is generally much less than those typically encountered in AKI [41].

There are also important limitations that exist in the published NGAL literature. The majority of the studies reported were from single centers that enrolled small numbers of subjects. Many studies did not report sensitivity, specificity, and AUCs for the diagnosis of AKI, parameters essential to determine the accuracy of any biomarker. The most important limitation of the studies is the inherent disadvantage of AKI definition that relies on the increased levels of serum creatinine. Large multicenter studies are required for further validation of its use in heterogeneous patient populations and for defining cutoff values [65].

## 4. Cystatin C

Cystatin C (Cys C) is a nonglycosylated protein with low molecular weight (13 kDa) belonging to the cystatin superfamily of cysteine endopeptidase (proteinases) inhibitors. Cysteine proteinases are enzymes that are involved in the intracellular catabolism of peptides and proteins. Cystatin C is a potent inhibitor of lysosomal proteinases and probably one of the most important extracellular inhibitors of cysteine proteases. It is produced by all nucleated cells of the body and released into the blood stream at a constant rate. Due to low molecular mass and absence of protein binding, cystatin C is freely filtered at the glomerulus and then reabsorbed by the proximal tubules, where it is catabolised [66]. Additionally it is not secreted by renal tubules. 

Cystatin C can be measured in a random sample of serum. All measures are based on liquid agglutination of latex particles coated with polyclonal antibodies against cystatin C. Cystatin C in the sample binds to anti-cystatin C antibody, which is coated on latex particles, and causes agglutination. The degree of the turbidity caused by agglutination can be measured optically and is proportional to the amount of cystatin C in the sample. There are two methods, depending on the nature of the signal measurement. Particle-enhanced turbidimetry immunoassay (PETIA) measures the transmitted light and Particle-enhanced nephelometric immunoassay (PENIA) measures the diffused light. Reference values may differ in many populations, with sex and age. Across different studies, the mean reference interval (as defined by the 5th and 95th percentiles) was between 0.52 and 0.98 mg/L [67]. For women, the average reference interval is 0.52 to 0.90 mg/L with a mean of 0.71 mg/L. For men, the average reference interval is 0.56 to 0.98 mg/L with a mean of 0.77 mg/L. The normal values decrease until the first year of life, remaining relatively stable before they increase again, especially beyond age 50. Because of its constant rate of production, serum cystatin C concentration is determined by glomerular filtration. Serum cystatin C is not diagnostically specific for AKI because it is an early marker of glomerular dysfunction rather than of tubular [68, 69].

The use of serum cystatin C against serum creatinine as an early detector of GFR impairment has been evaluated in several studies. Serum and urine cystatin C is not significally affected by nonrenal factors such as age or body mass [70]. Nevertheless, Knight et al. in a large cross-sectional study demonstrated that numerous nonrenal factors such as older age, male sex, obesity, smoking status, abnormal thyroid function, increased CRP, and use of immunosuppressive therapy (corticosteroids) can be associated with elevated serum cystatin C levels [71].

The ability of serum cystatin C to detect AKI in ICU population has been demonstrated with conflicting results. In a prospective study Herget-Rosenthal et al. measured serum creatinine and cystatin C daily in 85 patients at a high risk of developing AKI (defined by the RIFLE criteria). Forty-four patients developed AKI, and the increase of serum cystatin C preceded that of creatinine for 1-2 days [72]. Nejat et al. in univariate analysis of 318 ICU patients reported that serum cystatin C predicted developing sustained AKI with an AUC of 0.80 (95% confidence interval (CI) = 0.71–0.88) [73]. However, in a more recent multicenter prospective observational study in 151 ICU patients serum and urinary cystatin C were poor biomarkers for prediction of AKI (AUC = 0.72, no CI provided) [74].

The use of serum cystatin C for AKI prediction has been also studied in a subpopulation of ICU patients undergoing cardiac surgery. Haase-Fielitz et al. [17] measured concentrations of plasma NGAL, serum cystatin C, creatinine, and urea at baseline, on arrival in the ICU and at 24 h postoperatively in 100 adult cardiac surgical patients. On arrival in the ICU, plasma NGAL (AUC 0.80) and serum cystatin C (AUC 0.83) were of good predictive value, relative to creatinine (AUC 0.68) and urea (AUC 0.60). Plasma NGAL (AUC 0.95) and serum cystatin C (AUC 0.99) were also of excellent value in the prediction of adverse outcomes (need for RRT and in-hospital mortality).

In a recent prospective multicenter study Spahillari et al. compared the sensitivity and rapidity of AKI detection by serum cystatin C level relative to creatinine level after cardiac surgery. 1,150 high-risk adult cardiac surgery patients in the TRIBE-AKI (Translational Research Investigating Biomarker Endpoints for Acute Kidney Injury) Consortium participated. Overall, serum creatinine level detected more cases of AKI than cystatin C level: 35% developed a ≥25% increase in serum creatinine level, whereas only 23% had a ≥25% increase in cystatin C level (*P* < 0.001). However, confirmation by cystatin C level appeared to identify a subset of patients with AKI with a substantially higher risk of adverse outcomes [75]. 

As previously mentioned, cystatin C is completely reabsorbed by proximal tubules and does not experience circadian variation. Physiological urinary cystatin C concentrations are extremely low and can be measured by immunonephelometry on a random sample. It seems that reference values for freshly collected urine samples range from 0.03 to 0.18 mg/L [66, 76]. 

Any process that impairs renal tubules affects cystatin C reabsorption. Therefore, AKI can be associated with elevated urinary cystatin C levels [77, 78]. Particularly it appears that elevated urinary cystatin C levels are an indication of tubular dysfunction [76]. However, it was recently discovered that urinary secretion of cystatin C is augmented by albuminuria [79]. 

In a study of 444 ICU patients concentrations of urinary cystatin C were significantly higher in the presence of sepsis or AKI. Moreover urinary cystatin C had an AUC of 0.70 for the diagnosis of AKI. In this study urinary cystatin C was independently associated with AKI, sepsis, and death within 30 days [80].

In a cohort of 103 ICU patients undergoing cardiopulmonary bypass (CPB), urinary cystatin C had a moderate performance in predicting AKI 2 h after CPB (AUC = 0.72, CI 0.25–0.72) [81]. Another prospective study, performed by Koyner et al. in 123 patients undergoing cardiac surgery, evaluated the diagnostic utility of urinary NGAL, cystatin C, KIM-1, hepatocyte growth factor (HGF), *α*-GST (a proximal tubular damage marker), *π*-GST (a marker specific to distal tubule damage), FENa, and FEUrea as biomarkers for the detection of early and severe AKI after surgery. In this study, urine cystatin C upon admission best detected early stage 1 AKI (AUC = 0.70, CI 0.61–0.83) [82].

In a recent metaanalysis of 19 studies evaluating the predictive value of cystatin in diagnosing AKI in mixed ICU population (cardiac surgery, pediatric, and critically ill), serum cystatin C appeared to be a good biomarker in the prediction of AKI, whereas urinary cystatin C excretion had only moderate diagnostic value [83]. Specifically, across all settings of investigation, the diagnostic OR was 23.5 (95% CI, 14.2–38.9) for serum cystatin C level to predict AKI with sensitivity and specificity of 0.84 and 0.82, respectively. Nevertheless in the included studies, cutoff values varied across studies (0.8–2.04 mg/L) and the heterogeneity of the studied population was significant. A concise description of the function of Cystatin C in AKI is presented in Table 3.

## 5. KIM-1

Kidney injury molecule-1 (KIM-1 in humans and Kim-1 in rodents) is a type 1 transmembrane glycoprotein with an immunoglobulin and mucin domain. KIM-1 is also known as hepatitis A virus cellular receptor 1 and TIM-1, T cell immunoglobulin and mucin-containing molecule. Kim-1 gene was originally found to be most highly expressed 24–48 hours after ischemia in the rat kidney [84]. Normally KIM-1 protein is minimally expressed in kidney tissue or urine. The ectodomain segment of KIM-1 is shed from proximal tubules and can be detected in the urine by immunoassay [85]. Initially it was measured by direct sandwich enzyme-linked immunosorbent assay (ELISA) using monoclonal antibody and confirmed by western blot analysis. Recent studies utilized custom direct sandwich ELISA or microsphere-based Luminex xMAP technology using a commercially available polyclonal KIM-1 antibody. The soluble KIM-1 protein that appears in the urine of humans is 90 kDa. In the injured kidney KIM-1 is generated and accumulated at very high levels on the apical membrane of proximal tubules. In humans KIM-1 is upregulated in response to ischemic or nephrotoxic injury [84, 86, 87].

KIM-1 is believed to participate in the regeneration process after epithelial injury. It is also demonstrated that KIM-1 is a scavenger receptor on renal epithelial cell and plays an important role in the removal of dead cells from tubular lumen through phagocytosis [88].

KIM-1's utility as a biomarker for AKI was initially demonstrated in 2002. There was a markedly increase expression of KIM-1 in kidney biopsy specimens with confirmed acute tubular necrosis (ATN). In this study urinary level of KIM-1 was significantly higher in ischemic AKI compared with other causes of AKI (i.e., PRA, contrast-induced nephropathy) or chronic kidney disorder (CKD). Thus, KIM-1 may represent an early, noninvasive biomarker for proximal tubular AKI (Table 3).

Urinary KIM-1, along with N-acetyl-*β*-glucosaminidase (NAG) activity, was evaluated in 201 critically ill patients with AKI. Both urinary KIM-1 and NAG activity was associated with the degree of disease severity as determined by APACHE II and MOF scores. It was also suggested that urinary KIM-1 and NAG activity had a predictive value of adverse outcomes in patients with AKI (renal replacement therapy and hospital death) [89].

A prospective study was conducted in 90 adult cardiac surgery patients; 36 of whom developed AKI. The AUC to predict AKI immediately and 3 hours after surgery was 0.68 and 0.65 for urinary KIM-1. This study also demonstrated that combining KIM-1, N-acetyl-*β*-glucosaminidase (NAG), and NGAL enhanced the sensitivity of early detection of postoperative AKI [90]. Liangos et al. in a prospective study of 103 patients undergoing CPB investigated the performance of six urinary biomarkers for the early detection of AKI. Among the urinary biomarkers, KIM-1 had the better performance in predicting AKI 2 h after CPB (AUC: 0.78) [81].

The diagnostic and prognostic utility of novel and traditional AKI biomarkers was evaluated by Koyner et al. during a prospective study of 123 adults undergoing cardiac surgery. Preoperative KIM-1 was able to predict the future development of stage 1 and stage 3 AKI [82].

KIM-1 is highly expressed in acute tubular necrosis and in other clinical situations such as AKI postrenal transplantation, chronic kidney disease, and renal cell carcinoma. Available studies so far are insufficiently powered to establish a cutoff value that is predictive of AKI in the critical care setting. Most of available studies are relatively small, single-centered, and the heterogeneity of the studied population is significant. In some studies KIM-1 was combined with other urinary biomarkers for early detection of AKI in ICU population with moderate results.

## 6. IL-18

Interleukine-18 (IL-18) is a proinflammatory cytokine of the IL-1 superfamily. It is synthesized in an inactive form by several tissues including monocytes, macrophages, and proximal tubular epithelial cells. In animal models the role of IL-18 was demonstrated in postischemic AKI. Studies of isolated mouse proximal tubules demonstrated elevation of IL-18 following hypoxia, and mice with ischemic AKI had increased urinary levels of IL-18 [91, 92]. 

The ability of IL-18 to mediate ischemic proximal tubular injury in mice has led to the assumption that it can be used as an early biomarker of AKI in humans. Particularly its urine release has been explored as predictive of AKI in children undergoing cardiopulmonary bypass (CPB), adult receiving kidney transplant, and children requiring mechanical ventilation [59, 93, 94] (Table 3). IL-18 is measured through ELISA or a specific assay for their detection. 

The predictive value of IL-18 in critically ill patients was assessed in nested case-control study within the Acute Respiratory Distress Syndrome Network trial. Urine IL-18 levels >100 pg/mL were associated with increased odds of AKI of 6.5 (95% CI 2.1–20.4) in the next 24 hours. On a multivariable analysis urine IL-18 value on day 0 was an independent predictor of mortality [95].

In a prospective study, Siew et al. evaluated the capacity of urine IL-18 measured within 24 hours of intensive care unit (ICU) admission to predict AKI, death, and receipt of acute dialysis in a large mixed-adult ICU population. Of 451 patients, 86 developed AKI within 48 hours of enrolment. The overall predictive performance of urine IL-18 had AUC 0.62 (95% CI: 0.54 to 0.69). This value improved modestly to 0.67 (95% CI: 0.53 to 0.81) in patients whose enrolment eGFR was ≥75 mL/min per 1.73 m^2^. The highest median urine IL-18 levels were observed in patients with sepsis at enrolment. Additionally urine IL-18 remained independently predictive of poor clinical outcome (death or acute dialysis, odds ratio, 1.86 (95% CI: 1.31 to 2.64)) [96]. 

In a small pilot study of 55 patients receiving cardiopulmonary bypass, urine IL-18 was detected within 4–6 hours after surgery and peaked over 25-fold at 12 hours in the group of patients who eventually developed AKI. In the AKI group serum creatinine was raised to 48–72 h after CPB [93]. However, not all studies were able to demonstrate an adequate performance of urine IL-18 for the early detection of AKI. 

In a more recent single-centre study of 100 cardiac surgical patients, urine IL-18 did not appear to predict AKI during the postoperative period. In this study urine IL-18 correlated with the duration of cardiopulmonary bypass. 

## 7. L-FABP

Fatty-acid protein bindings (FABPs) are a family of 15-kDa cytoplasmatic proteins that are involved in the intracellular transport of long-chain fatty acids. They facilitate the transfer of fatty acids between extracellular and intracellular membranes. To date, nine different FABPs have been identified and named according to the tissues in which they were first identified. In addition, FABPs may also have a role in the reduction of cellular oxidative stress, binding fatty acid oxidation products, and limiting the toxic effects of oxidative intermediates on cellular membranes [97, 98]. 

L-FABP occurs mainly in the liver but, in small quantities, also in kidney and small intestine. Urinary L-FABP is undetectable in healthy control urine. L-FABP expression and urinary excretion were initially described in animal models of AKI. Under ischemic conditions the proximal tubular reabsorption of L-FABP is reduced [99, 100]. Urinary L-FABP is measured by enzyme-linked immunosorbent assay (ELISA). 

The performance of urinary L-FABP has been demonstrated in small series of ICU patients with promising results (Table 3). Urinary L-FABP was measured in 145 ICU patients with septic shock complicated with AKI. Urinary L-FABP at the time of admission was significantly higher in the nonsurvivors than in survivors with an AUC for mortality prediction of 0.99 [101]. In another small study of 25 ICU patients, 14 of who developed AKI, the diagnostic and predictive value of L-FABP was evaluated L-FABP at a cut-off value of 44.1 *μ*g/g Cr had an area under the curve 0.95 for the occurrence of AKI [102]. 

A recent study was performed to evaluate the performance of urinary L-FABP and NAG for AKI diagnosis in cardiac surgery patients. Of 77 patients, 28 patients developed AKI after surgery. Urinary L-FABP and NAG were significantly increased. However, receiver operating characteristic (ROC) analysis revealed that the biomarkers' performance was statistically significant but limited for clinical translation (area under the curve of ROC [AUC-ROC] for L-FABP at 4 hours 0.72 and NAG 0.75). Urinary L-FABP showed high sensitivity and NAG detected AKI with high specificity. When combined; these 2 biomarkers revealed that this combination panel can detect AKI with higher accuracy than either biomarker measurement alone (AUC-ROC 0.81) [103].

## 8. Conclusions

Acute kidney injury is a clinical situation with increased morbidity and mortality, especially among the ICU patients. Detection of AKI with current RIFLE and AKIN criteria is based on the increase in serum creatinine or decrease in urine output. Serum creatinine is unreliable and delayed marker of kidney damage. Serum creatinine becomes abnormal when more than 50% of GFR is lost, and it takes up to 24 hours before increases in blood concentration are detectable. New biomarkers, such as neutrophil gelatinase-associated lipocalin (NGAL), cystatin C (Cys C), kidney injury molecule-1 (KIM-1), interleukin-18 (IL-18) and liver-type fatty acid binding protein (L-FABP), seem to be more efficient in detecting AKI before the rise in serum creatinine. However various clinical studies of novel biomarkers have demonstrated moderate diagnostic accuracy. The majority of the studies reported were from single centers that enrolled small numbers of subjects and until now there are no determinant cutoff values for either of the new biomarkers. 

## Figures and Tables

**Table 1:** NGAL for prediction of AKI after cardiac surgery.

Reference	Publication year	No. of AKI pts	Type of NGAL	Sensitivity (%)	Specificity (%)	AUC-ROC
Mishra et al. [9]*	2005	20/71	pNGAL uNGAL	70.0 100.0	94.1 98.0	0.91 0.99
Wagener et al. [10]	2006	16/81	uNGAL	68.8	64.6	0.73
Dent et al. [11]*	2007	45/123	pNGAL	84.4	93.6	0.96
Wagener et al. [12]	2008	68/426	uNGAL	64.7	52.0	0.64
Bennett et al. [13]*	2008	99/196	uNGAL	78.8	91.8	0.95
Xin et al. [14]	2008	9/36	uNGAL	76.9	70.4	0.86
Koyner et al. [15]	2008	21/72 21/72	pNGAL uNGAL	44.4 66.7	75.9 64.8	0.56 0.68
Tuladhar et al. [16]	2009	9/50 9/50	pNGAL uNGAL	77.8 88.9	68.3 78.1	0.80 0.96
Haase-Fielitz et al. [17]	2009	23/100	sNGAL	78.3	77.9	0.80
Che et al. [18]	2010	14/29	uNGAL	84.0	80.0	0.85
Prabhu et al. [19]	2010	8/30	pNGAL	—	—	0.98
Perry et al. [20]	2010	75/879	pNGAL	38.7	81.5	0.64
Krawczeski et al. [21]*	2011	60/220	uNGAL	—	—	0.90
Parikh et al. [22]	2011	60/1219	uNGAL pNGAL	46 50	81 82	0.67 0.70
Fadel et al. [23]*	2012	19/40	pNGAL	100	90.5	0.95
Liu et al. [24]	2012	26/109	uNGAL	81.8	83.1	0.87

*Pediatric population, uNGAL: urine NGAL, pNGAL: plasma NGAL, sNGAL: serum NGAL, and AUC-ROC: area under the receiver-operating characteristic curve, —: not mentioned by the authors.

**Table 2:** NGAL for prediction of AKI in mixed ICU patients.

Reference	Publication year	No. of AKI pts	Type of NGAL	Sensitivity (%)	Specificity (%)	AUC-ROC
Zappitelli et al. [25]*	2007	16/39	uNGAL	75.0	69.6	0.76
Wheeler et al. [26]*	2008	22/143	sNGAL	86.4	38.8	0.68
Makris et al. [27]	2009	7/31	uNGAL	85.7	70.8	0.90
Siew et al. [28]	2009	86/451	uNGAL	60.0	80.0	0.71
Little et al. [29]	2009	161/604	uNGAL pNGAL	43.0 64.0	90.0 90.0	0.84 0.83
Cruz et al. [30]	2010	15/301	pNGAL	73.4	80.6	0.78
Shapiro et al. [31]	2010	13/558	pNGAL	78.0	80.0	0.84
Mårtensson et al. [32]	2010	18/45	uNGAL pNGAL	71.0 83.0	100.0 86.0	0.86 0.67
Constantin et al. [33]	2010	52/88	pNGAL	82.7	97.2	0.93
de Geus et al. [34]	2011	171/632	uNGAL pNGAL	89.0 82.0	70.0 70.0	0.80 0.77
Royakkers et al. [35]	2012	83/140	uNGAL sNGAL	— —	— —	0.48 0.53

*Pediatric population, uNGAL: urine NGAL, pNGAL: plasma NGAL, sNGAL: serum NGAL, and AUC-ROC: area under the receiver-operating characteristic curve, —: not mentioned by the authors.

**Table 3:** Properties of renal biomarkers.

Biomarker (sources)	Origin in AKI cases	Physiological function	Significance of rise	Studied clinical settings	Assay platform
NGAL (urine and circulating)	Urine: local synthesis in distal nephron in response to injury and secreted into urine Circulating: synthesized systemically in response to renal injury, filtered by glomerulus, and uptaken by proximal tubular cells with a little amount secreted in the urine	Bacteriostatic action, antioxidant effect, and growth and differentiation factor	Tubular injury (ischemia and nephrotoxins)	(i) Early detection of AKI after cardiac surgery in heterogeneous ICU population, in the emergency department, and after administration of nephrotoxins (ii) Risk stratification (iii) Prognostic marker after kidney transplantation (iv) Monitoring interventional trials in AKI (v) Prognosis of dialysis requirement in case of AKI establishment (vi) Prognosis of mortality	WB-ELISA-PETIA-POCT-CMIA

Cystatin C (urine and circulating)	Produced at a constant rate by nucleated cells, filtered by glomerulus, and almost completely reabsorbed in the proximal tubules	Inhibitor of lysosomal proteases and extracellular inhibitor of cysteine proteases	Change in GFR (proximal tubule injury)	(i) Early detection of AKI after cardiac surgery in heterogeneous ICU population and after administration of nephrotoxins (ii) Prognosis of mortality	PENIA-PETIA-ELISA

KIM-1 (urine)	Type 1 transmembrane protein that is highly expressed in dedifferentiated proximal tubule epithelial cells after ischemic or toxic injury and is not detectable in normal tissue	Regeneration process after epithelial injury, scavenger receptor for the removal of dead cells from tubular lumen through phagocytosis	Tubular injury (ischemia and nephrotoxins)	(i) Early detection of AKI after cardiac surgery and after administration of nephrotoxins (ii) Prognosis of dialysis requirement in case of AKI establishment (iii) Prognosis of mortality	WB-ELISA

IL-18 (urine)	Proinflammatory cytokineoriginates from tubular epithelial cells	Inflammation and immunomodulation mediator	Tubular injury (ischemia and nephrotoxins)	(i) Early detection of AKI after cardiac surgery in heterogeneous ICU population (ii) Prognostic marker after kidney transplantation (iii) Prognosis of mortality	ELISA

L-FABP (urine and circulating)	Production in the liver determines blood levels. Renal L-FABP is found in cytoplasm of the proximal tubules	Renal L-FABP helps maintain low levels of free fat acids in the cytoplasm	Tubular injury (ischemia and nephrotoxins)	(i) Early detection of AKI after cardiac surgery (ii) Prognosis of mortality	ELISA

ELISA: enzyme-linked immunosorbent assay, PENIA: particle-enhanced nephelometric immunoassay, PETIA: particle-enhanced turbidimetric immunoassay, WB: western blot, POCT: point-of-care test (triage), CMIA: chemiluminescent microparticle immunoassay ARCHITECT automated platform).
